# P11 A one-in-a-million case: familial cold autoinflammatory syndrome

**DOI:** 10.1093/rap/rkae117.042

**Published:** 2024-11-05

**Authors:** Sajeel Ahmed, Sana Azhar, Matthew Grove

**Affiliations:** Northumbria Healthcare NHS Foundation Trust, Cramlington, United Kingdom; South Tyneside and Sunderland NHS Foundation Trust, South Tyneside, United Kingdom; Northumbria Healthcare NHS Foundation Trust, Cramlington, United Kingdom

## Abstract

**Introduction:**

Familial cold autoinflammatory syndrome (FCAS) is a rare inherited inflammatory disorder characterised by episodes of rash, fever, arthralgia, and systemic inflammation triggered by exposure to cold. The onset occurs in early infancy and persists throughout life. The prevalence is less than 1 in 1,000,000. Diagnosis is primarily based on clinical presentation with genetic testing, specifically NLRP3 gene identification, used for confirmation. This case report describes a patient with FCAS who initially exhibited mild symptoms in early childhood, which later progressed to severe symptoms, including bilateral avascular necrosis of the hip.

**Case description:**

In 2019, a 69-year-old woman with no significant medical history presented to the rheumatology clinic with episodic inflammation of her extremities triggered by cold exposure, a condition she had experienced since childhood. These self-limiting episodes, typically lasting 24 hours, were characterised by painful, symmetrical swelling of the hands and livedo-like discoloration of the lower limbs.

Her symptoms first appeared in her late teens with Raynaud’s phenomenon, later developing into mucosal ulcers and sicca symptoms. Despite these manifestations, an immunology screen at the time was negative, and she did not meet the criteria for any autoimmune rheumatic disease. She continued to experience non-specific arthralgia and episodes of peripheral joint swelling when exposed to cold. These attacks spared her trunk, though she felt particularly unwell when consuming cold food, such as ice cream, suggesting possible internal attacks.

In 2012, she underwent genetic screening at the immunology center in Leeds, which was negative for NLRP3 and NLRP12 mutations. Consequently, she was diagnosed with an autoinflammatory-like syndrome based on clinical presentation. Treatment trials with hydroxychloroquine and anakinra provided no significant improvement, and anakinra was discontinued due to an allergic reaction.

By 2014, she reported bilateral hip pain. Imaging revealed osteoarthritic changes that rapidly progressed to avascular necrosis, necessitating bilateral hip replacement. That same year, she presented with shortness of breath and was prescribed furosemide. A CT chest scan revealed lymphoid interstitial pneumonia, which has since been monitored and remains stable.

Recently, she experienced worsening wrist pain. Nerve conduction studies confirmed bilateral carpal tunnel syndrome, which was treated with decompressive surgery. Repeat comprehensive immunology screening and bloodborne virus testing were negative. Microvascular imaging indicated delayed rewarming in the left hand, consistent with Raynaud’s phenomenon, and asymmetrical changes suggesting a more peripheral cause.

**Discussion:**

This case underscores the complexity of (FCAS, with the patient being evaluated by multiple specialists at different stages of her life. In light of possible Raynaud’s phenomenon and sicca symptoms, differentials such as Sjögren’s syndrome, acquired cold urticaria and other periodic fevers were considered, though diagnosis remained elusive, and negative genetic testing compounded the uncertainty. Notably, the genetics of inflammatory pathways are an area of active research. In this case, a pedigree analysis spanning three generations of affected and unaffected members revealed a clear autosomal dominant inheritance pattern. This family has been referred to a genetics centre for further research due to their interest.

It is likely that the current gene panel does not include the specific gene defect affecting this patient and her family. There have been instances of classic FCAS symptoms in patients without NLRP3 mutations, limiting the potential for targeted molecular treatments. Currently, there is no definitive treatment pathway for FCAS and management is primarily supportive, involving warming treatments and NSAIDs. Case reports have documented the use of high-dose corticosteroids and anakinra, a recombinant IL-1 receptor antagonist, with variable success. In this case, hydroxychloroquine provided some improvement in arthralgia and has been continued long-term with ophthalmic monitoring.

**Key learning points:**

• FCAS is caused by a mutation in the NLRP3 gene, which encodes cryopyrin on chromosome 1q44. This mutation activates the cryopyrin inflammasome, leading to uncontrolled inflammation through the inappropriate release of cytokines, including interleukin-1 beta (IL-1β). Cryopyrin is implicated in the inflammatory symptoms of FCAS and is associated with a spectrum of autoinflammatory disorders. NLRP3 mutations are also found in Muckle-Wells syndrome and chronic infantile neurologic cutaneous articular (CINCA) syndrome, which are considered different phenotypes of a single disease. In this particular case, multiple clinical features distinguished FCAS from other periodic fever syndromes. These included recurrent, intermittent episodes of fever and rash triggered by generalised cold exposure, an autosomal dominant inheritance pattern, brief episode durations typically less than 24 hours, and the notable absence of systemic manifestations such as deafness, periorbital edema, and serositis.

• Generalised cold exposure triggers FCAS symptoms. Although the exact mechanism of temperature dependence is still under investigation, monocytes in FCAS patients have shown enhanced IL-1 release at subnormal temperatures. Given the autosomal dominant inheritance pattern of FCAS, it is crucial to screen close family members, particularly first-degree relatives. Asymptomatic carriers in the family should consider genetic testing for early detection, aiding in timely diagnosis, patient reassurance, and management planning.

• The patient’s family members were not under specialist treatment and were advised to seek referral through their general practice. It is important to note that the genetic mutation may not always be present, highlighting the need for ongoing research in this area. Overall, this case underscores the varied presentations of this rare condition and the importance of a multidisciplinary team (MDT) approach in its management.

